# Supplementary material for: Language model-based B cell receptor sequence embeddings can effectively encode receptor specificity
Source: Nucleic Acids Res. 2023 Dec 18;52(2):548–57. doi: 10.1093/nar/gkad1128 (PMC10810273; doi:10.1093/nar/gkad1128)
Supplement: gkad1128_Supplemental_File [file gkad1128_supplemental_file.pdf]

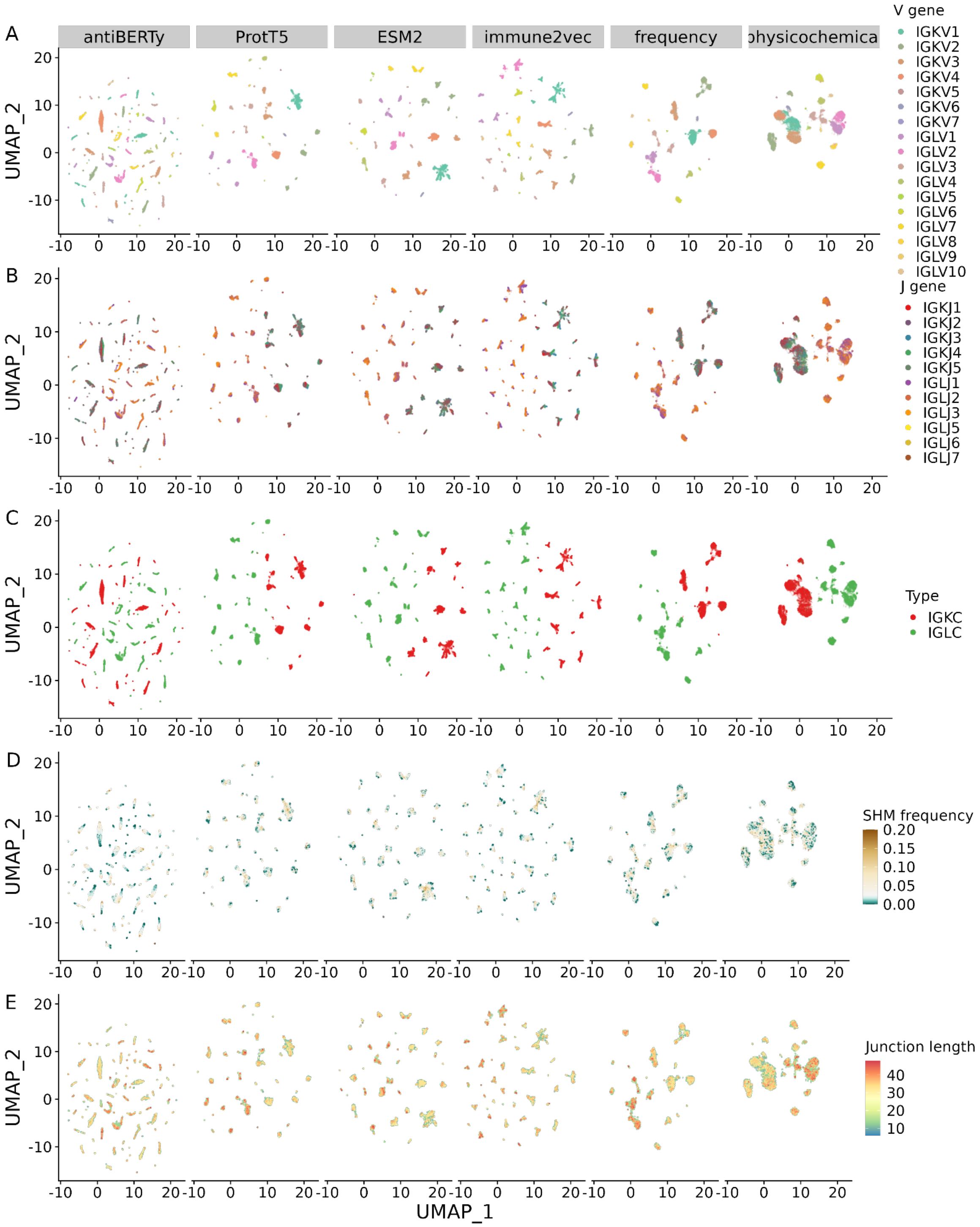

Figure S1. UMAP visualization of BCR light chain embeddings colored by (A) V gene family, (B) J gene family, (C) light chain type, (D) Somatic hypermutation frequency, (E) junction length.

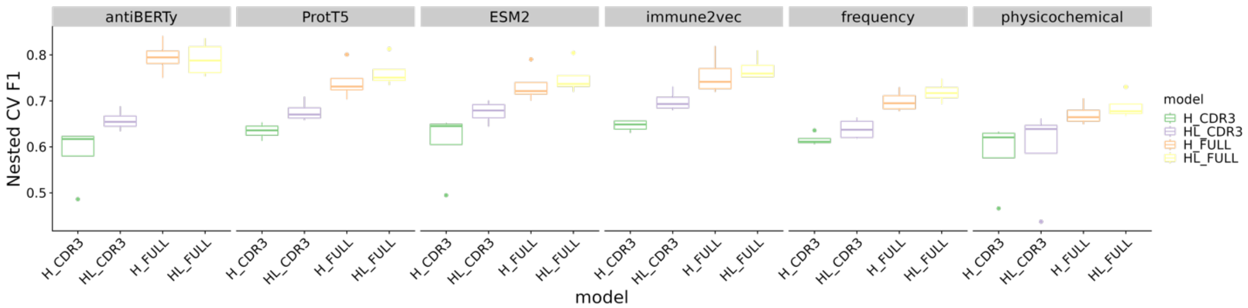

Figure S2. Receptor specificity prediction performance across embeddings and sequence inputs. H_CDR3: CDR3 heavy chain embedding, HL_CDR3: paired CDR3 heavy and light chain embedding concatenated, H_FULL: full-length heavy chain embedding, HL_FULL: paired full-length heavy and light chain embedding.

Task	Embedding	F1	MCC	ACC
Heavy Chain V Gene	antiBERTy	0.99901	0.99884	0.99901
Heavy Chain V Gene	ProtT5	0.99987	0.99985	0.99987
Heavy Chain V Gene	ESM2	0.99976	0.99971	0.99976
Heavy Chain V Gene	immune2vec	0.99991	0.9999	0.99991
Heavy Chain V Gene	frequency	0.97772	0.97379	0.97771
Heavy Chain V Gene	physicochemical	0.97603	0.97181	0.97602
Heavy Chain J Gene	antiBERTy	0.93527	0.92222	0.93515
Heavy Chain J Gene	ProtT5	0.9185	0.90192	0.91835
Heavy Chain J Gene	ESM2	0.91652	0.89979	0.91633
Heavy Chain J Gene	immune2vec	0.87761	0.8522	0.87579
Heavy Chain J Gene	frequency	0.60902	0.53365	0.6103
Heavy Chain J Gene	physicochemical	0.55156	0.46846	0.55574
Isotype	antiBERTy	0.5025	0.32273	0.50689
Isotype	ProtT5	0.48803	0.29502	0.48818
Isotype	ESM2	0.48162	0.28877	0.48172
Isotype	immune2vec	0.4902	0.3019	0.4917
Isotype	frequency	0.42697	0.21277	0.42684
Isotype	physicochemical	0.39883	0.17982	0.39836

Light Chain V Gene	antiBERTy	0.87559	0.87228	0.86947
Light Chain V Gene	ProtT5	0.87409	0.87211	0.86834
Light Chain V Gene	ESM2	0.86924	0.86787	0.86385
Light Chain V Gene	immune2vec	0.87079	0.86787	0.86469
Light Chain V Gene	frequency	0.84714	0.84016	0.83998
Light Chain V Gene	physicochemical	0.83482	0.82673	0.82793
Light Chain J Gene	antiBERTy	0.93427	0.92547	0.93427
Light Chain J Gene	ProtT5	0.93189	0.92294	0.93201
Light Chain J Gene	ESM2	0.92711	0.91779	0.92741
Light Chain J Gene	immune2vec	0.88107	0.86609	0.88179
Light Chain J Gene	frequency	0.70072	0.66166	0.70093
Light Chain J Gene	physicochemical	0.63796	0.59045	0.63679
Light Chain Type	antiBERTy	0.99984	0.79969	0.99984
Light Chain Type	ProtT5	1	0.8	1
Light Chain Type	ESM2	1	0.8	1
Light Chain Type	immune2vec	1	0.8	1
Light Chain Type	frequency	0.99691	0.79385	0.99692
Light Chain Type	physicochemical	0.99293	0.78587	0.99293

Table S1. Performance of BCR embeddings on sequence property classification tasks. Nest cross-validation was performed to evaluate the average weighted F1 score (F1), Matthew's correlation coefficient (MCC), and balanced accuracy (ACC) across the outer loops.

Task	Dimensionality	F1	MCC	ACC
Heavy Chain V Gene	25	0.99964	0.99957	0.99964
Heavy Chain V Gene	50	0.99988	0.99986	0.99988
Heavy Chain V Gene	100	0.99991	0.9999	0.99991
Heavy Chain V Gene	150	0.99991	0.9999	0.99991
Heavy Chain V Gene	200	0.99992	0.99991	0.99992
Heavy Chain V Gene	500	0.99991	0.99989	0.99991

Heavy Chain V Gene	1000	0.99998	0.99998	0.99998
Heavy Chain J Gene	25	0.70238	0.64558	0.70426
Heavy Chain J Gene	50	0.81401	0.77564	0.81192
Heavy Chain J Gene	100	0.87761	0.8522	0.87579
Heavy Chain J Gene	150	0.89795	0.8772	0.89726
Heavy Chain J Gene	200	0.91067	0.89219	0.90998
Heavy Chain J Gene	500	0.92328	0.90763	0.9228
Heavy Chain J Gene	1000	0.92885	0.91427	0.92852
Isotype	25	0.47532	0.28452	0.47687
Isotype	50	0.48663	0.29829	0.48801
Isotype	100	0.4902	0.3019	0.4917
Isotype	150	0.48122	0.29626	0.48429
Isotype	200	0.48703	0.29762	0.48912
Isotype	500	0.4867	0.29918	0.48737
Isotype	1000	0.48855	0.30091	0.48905
Light Chain V Gene	25	0.86612	0.85616	0.85717
Light Chain V Gene	50	0.86701	0.86078	0.85945
Light Chain V Gene	100	0.87079	0.86787	0.86469
Light Chain V Gene	150	0.87447	0.87092	0.86804
Light Chain V Gene	200	0.87713	0.87261	0.87021
Light Chain V Gene	500	0.88128	0.87614	0.87404
Light Chain V Gene	1000	0.8848	0.87821	0.87699
Light Chain J Gene	25	0.63304	0.59067	0.63793
Light Chain J Gene	50	0.79688	0.77137	0.79812
Light Chain J Gene	100	0.88107	0.86609	0.88179
Light Chain J Gene	150	0.90079	0.88799	0.90117
Light Chain J Gene	200	0.91214	0.90049	0.91222
Light Chain J Gene	500	0.9296	0.92019	0.92959

Light Chain J Gene	1000	0.93278	0.92403	0.93293
Light Chain Type	25	1	0.8	1
Light Chain Type	50	1	0.8	1
Light Chain Type	100	1	0.8	1
Light Chain Type	150	1	0.8	1
Light Chain Type	200	1	0.8	1
Light Chain Type	500	1	0.8	1
Light Chain Type	1000	1	0.8	1

Table S2. Changes in immune2vec embeddings performance with respect to dimensionality on sequence property classification tasks. Immune2vec models were trained with different dimensions (25 - 1000) to evaluate the effect of dimensionality on prediction performance. Nested cross-validation was performed to evaluate the average weighted F1 score (F1), Matthew's correlation coefficient (MCC), and balanced accuracy (ACC) across the outer loops.

Task	Embedding	RMSE	R2	MAE
Heavy Chain SHM frequency	antiBERTy	0.01789	0.91319	0.01294
Heavy Chain SHM frequency	ProtT5	0.02445	0.83653	0.01861
Heavy Chain SHM frequency	ESM2	0.02296	0.85567	0.0174
Heavy Chain SHM frequency	immune2vec	0.02313	0.85605	0.01765
Heavy Chain SHM frequency	frequency	0.04842	0.37989	0.03937
Heavy Chain SHM frequency	physicochemical	0.04838	0.38469	0.03919
Heavy Chain Junction Length	antiBERTy	19.6459	-5.8229	17.06233
Heavy Chain Junction Length	ProtT5	19.38826	-0.20392	17.05321
Heavy Chain Junction Length	ESM2	19.3443	-6.23156	17.22838
Heavy Chain Junction Length	immune2vec	23.02751	-9.02568	20.04956
Heavy Chain Junction Length	frequency	26.35413	-11.23459	23.34738
Heavy Chain Junction Length	physicochemical	26.07709	-1.19957	22.96459
Light Chain SHM frequency	antiBERTy	0.01691	0.9075	0.01257
Light Chain SHM frequency	ProtT5	0.0226	0.83667	0.01725

Light Chain SHM frequency	ESM2	0.02155	0.8501	0.0163
Light Chain SHM frequency	immune2vec	0.02459	0.80612	0.0185
Light Chain SHM frequency	frequency	0.04976	0.25127	0.03907
Light Chain SHM frequency	physicochemical	0.04946	0.25512	0.03887
Light Chain Junction Length	antiBERTy	11.84322	-0.87715	10.39247
Light Chain Junction Length	ProtT5	11.26954	-0.75678	9.76896
Light Chain Junction Length	ESM2	11.13957	-0.78333	9.69484
Light Chain Junction Length	immune2vec	13.09683	-1.39173	11.95008
Light Chain Junction Length	frequency	13.69778	-1.5855	12.68878
Light Chain Junction Length	physicochemical	13.74507	-1.75419	12.75006

Table S3. Performance of BCR embeddings on sequence property regression tasks. Nest cross-validation was performed to evaluate the average root mean square error (RMSE), adjusted R2 (R2), and mean absolute error (MAE) across the outer loops.

Task	Dimensionality	RMSE	R2	MAE
Heavy Chain SHM frequency	25	0.0262	0.81669	0.02016
Heavy Chain SHM frequency	50	0.02387	0.84633	0.01815
Heavy Chain SHM frequency	100	0.02313	0.85605	0.01765
Heavy Chain SHM frequency	150	0.02264	0.86081	0.01726
Heavy Chain SHM frequency	200	0.02263	0.86278	0.01722
Heavy Chain SHM frequency	500	0.02177	0.87329	0.01644
Heavy Chain SHM frequency	1000	0.02182	0.87219	0.01645
Heavy Chain Junction Length	25	24.102	-9.78745	21.1315
Heavy Chain Junction Length	50	23.39717	-9.14411	20.36449
Heavy Chain Junction Length	100	23.02751	-9.02568	20.04956
Heavy Chain Junction Length	150	22.85182	-7.77051	19.75448
Heavy Chain Junction Length	200	22.85676	-8.051	19.8138
Heavy Chain Junction Length	500	21.97517	-0.54174	18.83146
Heavy Chain Junction Length	1000	22.3864	-7.71062	19.2307

Light Chain SHM frequency	25	0.03126	0.69164	0.02395
Light Chain SHM frequency	50	0.02735	0.75988	0.02061
Light Chain SHM frequency	100	0.02459	0.80612	0.0185
Light Chain SHM frequency	150	0.02236	0.83817	0.01683
Light Chain SHM frequency	200	0.02158	0.85161	0.01624
Light Chain SHM frequency	500	0.02049	0.86538	0.01534
Light Chain SHM frequency	1000	0.02029	0.86732	0.01514
Light Chain Junction Length	25	13.85732	-1.85679	12.9124
Light Chain Junction Length	50	13.43479	-1.61517	12.38213
Light Chain Junction Length	100	13.09683	-1.39173	11.95008
Light Chain Junction Length	150	12.9498	-1.33768	11.7698
Light Chain Junction Length	200	12.85761	-1.25761	11.65768
Light Chain Junction Length	500	12.85821	-1.35267	11.60367
Light Chain Junction Length	1000	12.90942	-1.52857	11.83083

Table S4. Changes in immune2vec embeddings performance with respect to dimensionality on sequence property regression tasks. Immune2vec models were trained with different dimensions (25 - 1000) to evaluate the effect of dimensionality on prediction performance. Nested cross-validation was performed to evaluate the average root mean square error (RMSE), adjusted R2 (R2), and mean absolute error (MAE) across the outer loops.

Embedding	Model	Sequence	F1	MCC	ACC	AUROC
antiBERTy	HL	FULL	0.79122	0.46727	0.74111	0.74111
antiBERTy	HL	CDR3	0.65778	0.26267	0.64794	0.64794
antiBERTy	H	FULL	0.7952	0.45564	0.73557	0.73557
antiBERTy	H	CDR3	0.58582	0.14125	0.57883	0.57883
ProtT5	HL	FULL	0.76205	0.41351	0.72184	0.72184
ProtT5	HL	CDR3	0.67696	0.27244	0.65228	0.65228
ProtT5	H	FULL	0.74161	0.3661	0.69588	0.69588
ProtT5	H	CDR3	0.63451	0.19168	0.60829	0.60829
ESM2	HL	FULL	0.74949	0.38407	0.70602	0.70602

ESM2	HL	CDR3	0.67587	0.26867	0.65034	0.65034
ESM2	H	FULL	0.73344	0.34726	0.68567	0.68567
ESM2	H	CDR3	0.60926	0.17603	0.59803	0.59803
immune2vec	HL	FULL	0.77024	0.41612	0.72064	0.72064
immune2vec	HL	CDR3	0.69919	0.29799	0.66538	0.66538
immune2vec	H	FULL	0.7554	0.38084	0.70213	0.70213
immune2vec	H	CDR3	0.64611	0.19681	0.61062	0.61062
frequency	HL	FULL	0.71882	0.32801	0.67879	0.67879
frequency	HL	CDR3	0.63892	0.2082	0.61718	0.61718
frequency	H	FULL	0.69916	0.28339	0.65464	0.65464
frequency	H	CDR3	0.61605	0.12876	0.57148	0.57148
physicochemical	HL	FULL	0.68823	0.26771	0.64583	0.64583
physicochemical	HL	CDR3	0.59417	0.16532	0.59236	0.59236
physicochemical	H	FULL	0.67093	0.23082	0.62642	0.62642
physicochemical	H	CDR3	0.58512	0.121	0.56746	0.56746

Table S5. Performance of BCR embeddings on receptor specificity prediction tasks. Nest cross-validation was performed to evaluate the average weighted F1 score (F1), Matthew's correlation coefficient (MCC), balanced accuracy (ACC), and area under the receiver operating characteristics (AUROC) across the outer loops.

Dimensionality	Model	Sequence	F1	MCC	ACC	AUROC
25	HL	FULL	0.74485	0.36293	0.6942	0.6942
25	HL	CDR3	0.68858	0.27881	0.65513	0.65513
25	H	FULL	0.73577	0.34113	0.68307	0.68307
25	H	CDR3	0.64503	0.17744	0.59878	0.59878
50	HL	FULL	0.7602	0.39052	0.70657	0.70657
50	HL	CDR3	0.68074	0.27538	0.65382	0.65382
50	H	FULL	0.7492	0.36449	0.69366	0.69366
50	H	CDR3	0.64911	0.19428	0.6091	0.6091

100	HL	FULL	0.77024	0.41612	0.72064	0.72064
100	HL	CDR3	0.69919	0.29799	0.66538	0.66538
100	H	FULL	0.7554	0.38084	0.70213	0.70213
100	H	CDR3	0.64611	0.19681	0.61062	0.61062
150	HL	FULL	0.76938	0.41248	0.71807	0.71807
150	HL	CDR3	0.70371	0.30946	0.67176	0.67176
150	H	FULL	0.75912	0.38889	0.70594	0.70594
150	H	CDR3	0.64305	0.19791	0.61146	0.61146
200	HL	FULL	0.77004	0.41893	0.72268	0.72268
200	HL	CDR3	0.69318	0.29069	0.66194	0.66194
200	H	FULL	0.75994	0.39183	0.708	0.708
200	H	CDR3	0.63477	0.18228	0.60228	0.60228
500	HL	FULL	0.76096	0.40433	0.71623	0.71623
500	HL	CDR3	0.6965	0.30191	0.66825	0.66825
500	H	FULL	0.75667	0.39011	0.70779	0.70779
500	H	CDR3	0.62783	0.17731	0.59921	0.59921
1000	HL	FULL	0.76128	0.40904	0.7196	0.7196
1000	HL	CDR3	0.69282	0.29736	0.66579	0.66579
1000	H	FULL	0.74906	0.37547	0.70085	0.70085
1000	H	CDR3	0.60506	0.15641	0.58631	0.58631

Table S6. Changes in immune2vec embeddings performance with respect to dimensionality on receptor specificity prediction tasks. Immune2vec models were trained with different dimensions (25 - 1000) and different sequence inputs to evaluate the effect of dimensionality on prediction performance. Nested cross-validation was performed to evaluate the average weighted F1 score (F1), Matthew's correlation coefficient (MCC), balanced accuracy (ACC), and area under the receiver operating characteristics (AUROC) across the outer loops.
